# Post-retirement mental health: Risk factors and preventive strategies for isolation and depression

**DOI:** 10.1192/j.eurpsy.2026.11580

**Published:** 2026-07-31

**Authors:** C. Sridi, I. Kacem, J. Ben Khelifa, R. Nakhli, F. Chelly, N. Belhadj, N. Gannoun, A. Fki, M. Maoua

**Affiliations:** 1 Occupational Medicine Department, Sahloul University Hospital; 2 University of Sousse, Faculty of Medicine of Sousse; 3 Research Laboratory LR19SP03; 4 Occupational Medicine Department, Farhat Hached University Hospital; 5 Clinical Psychology Unit of Sahloul University Hospital, Sousse, Tunisia

## Abstract

**Introduction:**

The transition to retirement, far from being a mere cessation of work, is a major life and psychosocial transition that can negatively affect mental health. Social isolation and depression are well-established risks during this period. Understanding their determinants is essential for developing effective preventive measures.

**Objectives:**

This literature review aims to identify the key risk factors for deterioration of mental health after retirement and to synthesize validated strategies for prevention.

**Methods:**

A review of the literature was conducted using PubMed, Scopus, and Google Scholar for publications between 2014 and 2024. Keywords included “retirement”, “mental health”, “depression”, “social isolation”, “risk factors”, “preventive strategies”. Included studies were meta-analyses, systematic reviews, and longitudinal studies that assessed predictors of depression or isolation in recent retirees, as well as the effectiveness of preventive interventions.

**Results:**

The literature consistently identifies several major risk factors. Individuals most vulnerable to depression and social isolation after retirement are those with:

- Involuntary retirement: Leaving work due to health problems or financial necessity.
- Poor preparation: Lack of anticipation and absence of a post-professional life plan.
- Strong professional identity: Work being the main source of social status and interpersonal interactions.
- Limited social network: Few meaningful connections outside the workplace.

Effective preventive strategies target these factors and include multidimensional retirement preparation programs, support for social and volunteer engagement, and early identification and intervention by healthcare professionals.

**Conclusions:**

Prevention of post-retirement psychological distress requires a dual approach: early identification of risk factors and implementation of targeted preventive strategies. Proactive interventions before and during the transition to retirement can transform this potentially vulnerable period into a rewarding phase of life. Occupational physicians upstream and attending physicians downstream play critical roles in this preventive continuum.

**Disclosure of Interest:**

None Declared

